# A Rare Malignant Peripheral Nerve Sheath Tumor of the Maxilla Mimicking a Periapical Lesion

**DOI:** 10.1155/2016/4101423

**Published:** 2016-11-23

**Authors:** José Alcides Arruda, Pamella Álvares, Luciano Silva, Alexandrino Pereira dos Santos Neto, Cleomar Donizeth Rodrigues, Antônio Caubi, Marcia Silveira, Sandra Sayão, Ana Paula Sobral

**Affiliations:** ^1^Faculdade de Odontologia de Pernambuco, Universidade de Pernambuco, Avenida General Newton Cavalcante, 1650 Aldeia dos Camarás, 54.753-020 Camaragibe, PE, Brazil; ^2^Faculdades Integradas da União Educacional do Planalto Central, SIGA Área Especial para Indústria, n^o^ 2, Setor Leste, 72.445-020, Gama, DG, Brazil

## Abstract

Malignant peripheral nerve sheath tumor is a malignant neoplasm that is rarely found in the oral cavity. About 50% of this tumor occurs in patients with neurofibromatosis type I and comprises approximately 10% of all soft tissue sarcomas of head and neck region. Intraosseous malignant peripheral nerve sheath tumor of the maxilla is rare. This article is the first to address malignant peripheral nerve sheath tumor of the maxilla presenting as a periapical radiolucency on nonvital endodontically treated teeth in the English medical literature. Surgical approaches to malignant soft tissue tumor vary based on the extent of the disease, age of the patient, and pathological findings. A rare case of intraosseous malignant peripheral nerve sheath tumor is reported in a 16-year-old woman. The patient presented clinically with a pain involving the upper left incisors region and with defined unilocular periapical radiolucency lesion involved between the upper left incisors. An incisional biopsy was made. Histological and immunohistochemical examination were positive for S-100 protein and glial fibrillary acidic protein showed that the lesion was an intraosseous malignant peripheral nerve sheath tumor of the maxilla. Nine years after the surgery, no regional recurrence was observed.

## 1. Introduction

Malignant peripheral nerve sheath tumor (MPNST) is a malignant neoplasm that is rarely found in the oral cavity. About 50% of this tumor occurs in patients with neurofibromatosis type 1 (NF-1) and comprises approximately 10% of all soft tissue sarcomas of both head and neck region [1]. Its incidence in the oral region is extremely low, roughly 0,001% [2]. Its occurrence in head and neck regions involves soft and hard tissues [3]. However, intraosseous maxillary MPNST presenting as a periapical lesion is exceptionally unusual. This tumor type occurs in peripheral nerves and may exhibit differentiation in nerve sheath elements such as Schwann cells and perineural cells, as well as fibroblasts [1]. The differential diagnosis of this neoplasm may be facilitated by additional information obtained from immunohistochemistry findings [2].

This article reports the unusual clinical case of intraosseous MPNST with no association with NF-1, presenting as a periapical lesion on nonvital endodontically treated teeth.

## 2. Case Presentation

**Figure 1:**
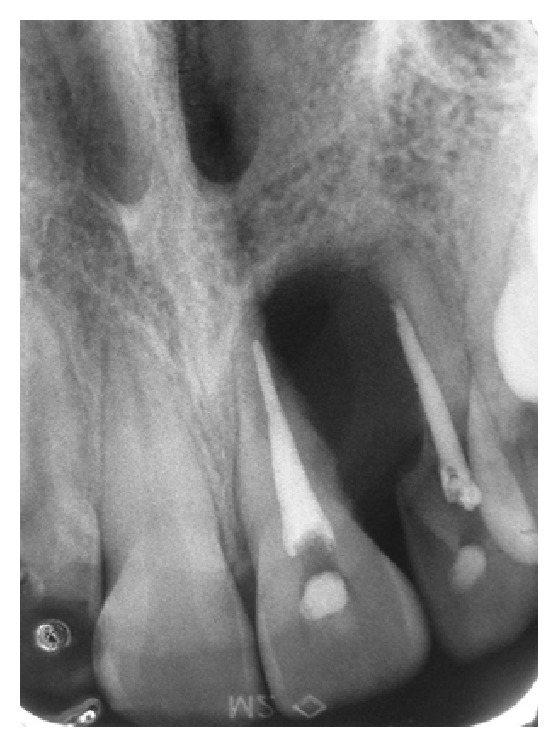
Periapical radiograph showing radiopaque image of the roots of teeth 21 and 22, which are compatible with root canal filling material, obtained from a radiolucent image of the blurred boundaries in the region of the upper incisors.

**Figure 2:**
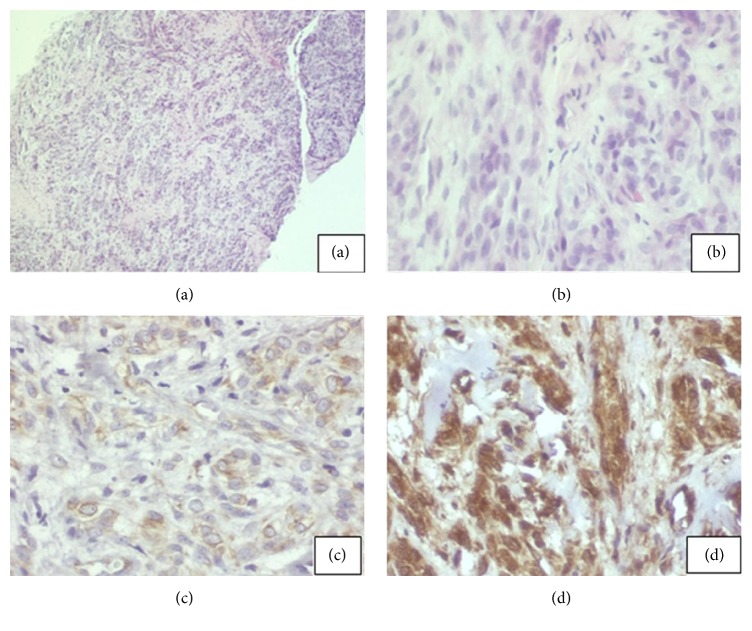
(a) Cell bundle arrangements with rounded, large nuclei that sometimes contain palisades, strands, and/or hyalinized islands. Hematoxylin and eosin (HE) staining, 40x. (b) Spindle cells with comma-shaped nuclei. HE staining, 100x. (c) Cells positive for S-100 protein. IHC, 200x. (d) Cells positive for glial fibrillary acidic protein (GFAP). IHC, 200x.

**Figure 3:**
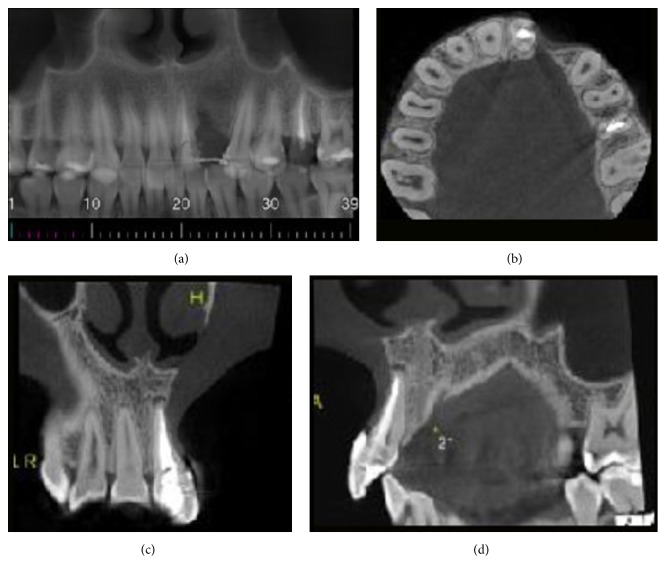
(a) CT panoramic reconstruction of maxilla. (b) CT axial reconstruction of the jaw. (c) CT coronal reconstruction of anterior region of maxilla. (d) CT sagittal reconstruction of tooth 11 region.

A 16-year-old female was referred with pain involving the upper left incisors region. Patient history revealed endodontic treatment of the maxillary upper left incisors with necrotic pulp and periapical radiolucency six months earlier. A few months after the treatment, she experienced discomfort pain. Extraoral examination revealed an integrate mucosa with normal appearance, whereas, intraorally, the upper left lateral incisor presented chromatic alterations and mobility in the region. A periapical radiograph showed the presence of a defined radiolucency lesion between the upper left incisors of the endodontically treated teeth with a size of 2.5 × 1.0 cm in a high diameter (Figure 1). An incisional biopsy was made using local anesthesia and sent to the Oral Pathology Laboratory. The specimen was fixed in 10% neutral formalin and routinely prepared for light microscopy; the sections were stained with hematoxylin and eosin. Histological examination showed malignant neoplasm fragments consisting of fusiform cells with comma-shaped nuclei, originating from cell bundles exhibiting one of two forms: a round shape with large nuclei (occasionally palisading) or hyalinized strands and/or islets (Figures 2(a) and 2(b)). Immunohistochemistry analysis was performed using streptavidin-biotin technique with monoclonal antibodies; the cells were positive for both the S-100 protein and the glial fibrillary acidic protein (GFAP) (Figures 2(c) and 2(d)). The immunohistochemical expression of protein S-100 was weak at less than 50% of the tumor cells (Figure 2(c)), and a diagnosis of MPNST of the maxilla was made. The patient was referred to the Oncology Department and was subjected to surgical excision of the upper left lateral incisor as well as the total removal of the remaining lesion and adjuvant chemotherapy. The examination of the surgical part had not exhibited banks committed and it was staged as low grade of malignancy. The research of metastasis through the exams for images revealed no secondary tumors. The conduct of not using radiotherapy was a decision of the Clinical Oncology team. The patient was rehabilitated with an adhesive prosthesis in the region of the removal tooth (Figure 3). In the last exam, cone beam computerized tomography (CBCT) imaging examination revealed the absence of the upper left lateral incisor, a loss of bone matter, and the absence of vestibular and lingual cortices in the inferior and central thirds of the alveolar ridge (vide reconstructions: panoramic in Figure 3(a) and axial in Figure 3(b)). The tomography revealed normal trabeculation of adjacent bone tissue and preservation of the upper third of the alveolar ridge. The upper left central incisor exhibited a filled canal and a hypodense periapical area, suggestive of an inflammatory periapical lesion or a bone repair lesion after endodontic treatment. The absence of alveolar lamina dura and the loss of radicular matter in the mesiobuccal face were consistent with external root resorption and/or surgical resection (vide reconstructions: coronal in Figure 3(c) and upper left lateral incisor tooth in Figure 3(d) (sagittal)). The patient was tumor-free at the nine-year follow-up consultation.

## 3. Discussion

This paper describes an unusual case of intraosseous maxillary MPNST mimicking a periapical lesion in order to call attention of clinicians to the fact that several different diseases are able to mimic endodontic periapical lesions. The available literature shows that there are no reports of this tumor presenting as periapical lesion in the upper left incisors. It also shows the complexity of a diagnosis of rare bone tumors when based on clinical and radiographic features only. Besides, MPNST onset occurs in a peripheral nerve that may exhibit differentiation of nerve sheath elements, which is more common in the proximal extremities and stem of the nerve [4]. Although uncommon, occurrence in cranial nerves usually targets the fiftieth cranial nerve pair [1].

Patient history, clinical examinations, pulp sensitivity tests, and endodontic treatment follow-up are essential for the diagnosis of inflammatory periapical lesions. The presence of periapical images and dental resorption are characteristic of endodontic and/or odontogenic origin lesions. Soft tissue tumor diagnoses are extremely difficult, and diagnosis of MPNST is particularly difficult because it lacks diagnostic criteria. Although no consensus exists regarding the classification of sarcomas as a type of MPNST, the reported lesion could be placed in this category because its onset occurred in a peripheral nerve or neurofibroma, and it fulfilled one of the three established diagnosis criteria, which include (1) the tumor originating in a peripheral nerve that does not exhibit lines of aberrant or heterologous differentiation; (2) the tumor originating from a preexisting benign nerve sheath tumor, usually a neurofibroma; or (3) the tumor exhibiting characteristics that are commonly observed in tumors originating from the aforementioned situations or malignant Schwann cell tumors [2, 3]. The present case fulfills the diagnostic criterion of MPNST onset occurring in a peripheral nerve.

Despite imaging features of MPNST being nonspecific and noncharacteristic, the role of radiologic diagnosis is to ensure that MPNST are preoperatively suggested as a diagnosis and are differentiated from malignant lesions through a series of specific imaging findings. It is important to highlight the aggressiveness of the lesion by a radiographic examination, once it is required to analyze bone destruction, extra bone invasion, structure of the lesion, and root resorption [5].

Radiographic findings of intraosseous MPNST may show a great variation, from unilocular to multilocular, with or without well-defined borders of the lesion, and cortical expansion. Periapical radiographs of the presenting case of intraosseous MPNST showed a unilocular periapical lesion with a defined border and external dental resorption in the lateral region and apical region of upper left incisors. This radiographic appearance was suggestive of a periapical lesion of cyst, once external root resorption does not occur exclusively of malignancies, as what may occur for orthodontic movement, neoplasia, being more common in case of inflammatory origin, such as periapical lesion [5].

Establishing a diagnosis requires immunohistochemistry technique because MPNST may be similar to fibrosarcoma. And a plethora of other bone tumors are included in differential diagnoses such as central giant cell lesion, fibrous dysplasia, ameloblastoma, myxoma, haemangioma, neurofibroma, leiomyoma, sarcoma, or simply periapical lesions [1]. It is also essential to include S-100 protein for suspected neural tumors, since it is positive in all neural tumors [6]. And the use of GFAP refers to filament proteins type III intermediate and it is the essential component of the cytoskeleton in astrocytes of all vertebrates [7]. However, the latter exhibits irregularly shaped cells. These tumors also exhibit characteristics such as regular Schwann cells, with dense and hypodense fascicles creating a marbleized effect and myxoid areas; elongated, asymmetric cone-shaped cells with irregular, palisading, and comma- or buckle-shaped nuclei; spiral structures; peculiar hyperplastic perivascular alterations; and, occasionally, heterologous elements (cartilage, bone, and muscle, which are present in 10–15% of tumors). These tumors may exhibit a greater degree of cellular differentiation, pleomorphism, and mitotic activity [3].

Malignant peripheral nerve sheath tumors were most commonly located on the extremities (45%), then trunk (34%), and head and neck region (19%) [8]. In head and neck region, they occur most frequently in both nasopharynx and the nasal cavity [9]. The absence of such reports confirms the difficulty of performing differential diagnoses of periapical pathologies because tumors can mimic an inflammatory periapical lesion. The recommended treatment option for MPNST is surgical excision. However, it is a difficult procedure due to poor anatomical accessibility and its probability of recurrence varies approximately between 40 and 65% in head and neck region and approximately between 40 and 68% elsewhere [4]. Surgical approaches to malignant soft tissue tumor vary based on the extent of the disease, age of the patient, and pathological findings. Yet, surgical excision with wide margins is the first treatment choice for the majority of patients. Radiation and/or chemotherapy may also be used due to the high recurrence rates or in the case of an incomplete lesion excision. Adjuvant radiation and/or chemotherapy treatment must always be considered and guided by the clinical stage [10, 11]. Nonetheless, in the case reported, the patient opted for surgical excision in conjunction with chemotherapy. The patient responded well to the treatment and was disease-free at the nine-year control follow-up evaluation.

To establish the diagnoses of MPNST, it requires immunohistochemistry technique. Radiographically, maxillary intraosseous MPNST is difficult to differentiate from other bone lesions. The differential diagnosis of the case presented in this article included periapical inflammatory lesions, such as periapical granuloma or cyst. The root resorption is not a usual finding of the above lesions. Root resorption can be seen in central giant cell granuloma or an odontogenic neoplasm such as ameloblastoma or in malignant tumors. Nine years after the surgery, no regional recurrence was observed.

## References

[1] Schaefer I.-M., Fletcher C. D. M. (2015). Malignant peripheral nerve sheath tumor (MPNST) arising in diffuse-type neurofibroma: clinicopathologic characterization in a series of 9 cases. *The American Journal of Surgical Pathology*.

[2] Zou C., Smith K. D., Liu J. (2009). Clinical, pathological, and molecular variables predictive of malignant peripheral nerve sheath tumor outcome. *Annals of Surgery*.

[3] Baehring J. M., Betensky R. A., Batchelor T. T. (2003). Malignant peripheral nerve sheath tumor: the clinical spectrum and outcome of treatment. *Neurology*.

[4] Ramalingam W. V. B. S., Nair S., Mandal G. (2012). Malignant peripheral nerve sheath tumor of the oral cavity. *Journal of Oral and Maxillofacial Surgery*.

[5] Consolaro A., Furquim L. Z. (2014). Extreme root resorption associated with induced tooth movement: a protocol for clinical management. *Dental Press Journal of Orthodontics*.

[6] Chrysomali E., Papanicolaou S. I., Dekker N. P., Regezi J. A. (1997). Benign neural tumors of the oral cavity: a comparative immunohistochemical study. *Oral Surgery, Oral Medicine, Oral Pathology, Oral Radiology, and Endodontics*.

[7] Sukhorukova E. G., Kruzhevskiĭ D. É., Alekseeva O. S. (2015). Glial fibrillary acidic protein: the component of intermediate filaments in the vertebrate brain astrocytes. *Zhurnal Evoliutsionnoĭ Biokhimii i Fiziologii*.

[8] Meshikhes A. N., Duhaileb M. A., Amr S. S. (2016). Malignant peripheral nerve sheath tumor with extensive osteosarcomatous and chondrosarcomatous differentiation: a case report. *International Journal of Surgery Case Reports*.

[9] Stucky C.-C. H., Johnson K. N., Gray R. J. (2012). Malignant peripheral nerve sheath tumors (MPNST): the Mayo Clinic experience. *Annals of Surgical Oncology*.

[10] Kar M., Deo S. V., Shukla N. K. (2006). Malignant peripheral nerve sheath tumors (MPNST)—clinicopathological study and treatment outcome of twenty-four cases. *World Journal of Surgical Oncology*.

[11] Ziadi A., Saliba I. (2010). Malignant peripheral nerve sheath tumor of intracranial nerve: a case series review. *Auris Nasus Larynx*.

